# Human Granulocytic Anaplasmosis and *Anaplasma phagocytophilum*

**DOI:** 10.3201/eid1112.050898

**Published:** 2005-12

**Authors:** J. Stephen Dumler, Kyoung-Seong Choi, Jose Carlos Garcia-Garcia, Nicole S. Barat, Diana G. Scorpio, Justin W. Garyu, Dennis J. Grab, Johan S. Bakken

**Affiliations:** * Johns Hopkins University School of Medicine, Baltimore, Maryland, USA; † University of Minnesota at Duluth, Duluth, Minnesota, USA; ‡ St. Luke's Hospital, Duluth, Minnesota, USA

**Keywords:** Anaplasma phagocytophilum, Anaplasmataceae, Rickettsiales, Tickborne Diseases, Communicable Diseases, Emerging, Neutrophils, Inflammation, research

## Abstract

Human granulocytic anaplasmosis is a tickborne rickettsial infection of neutrophils caused by *Anaplasma phagocytophilum*. The human disease was first identified in 1990, although the pathogen was defined as a veterinary agent in 1932. Since 1990, US cases have markedly increased, and infections are now recognized in Europe. A high international seroprevalence suggests infection is widespread but unrecognized. The niche for *A*. *phagocytophilum*, the neutrophil, indicates that the pathogen has unique adaptations and pathogenetic mechanisms. Intensive study has demonstrated interactions with host-cell signal transduction and possibly eukaryotic transcription. This interaction leads to permutations of neutrophil function and could permit immunopathologic changes, severe disease, and opportunistic infections. More study is needed to define the immunology and pathogenetic mechanisms and to understand why severe disease develops in some persons and why some animals become long-term permissive reservoir hosts.

## Abstract 2

Understanding how *Anaplasma phagocytophilum* alters neutrophils will improve diagnosis, treatment, and prevention of this severe illness.

Human granulocytic anaplasmosis (HGA) was first identified in 1990 in a Wisconsin patient who died with a severe febrile illness 2 weeks after a tick bite (*1*). During the terminal phases of the infection, clusters of small bacteria were noted within neutrophils in the peripheral blood (Figure 1), assumed to be phagocytosed gram-positive cocci. A careful review of the blood smear suggested the possibility of human ehrlichiosis, an emerging infection with similar bacterial clusters in peripheral blood monocytes among infected patients in the southeast and south-central United States. All blood cultures were unrevealing, and specific serologic and immunohistochemical tests for *Ehrlichia chaffeensis*, the causative agent of human monocytic ehrlichiosis (HME) were negative. Over the ensuing 2 years, 13 cases with similar intraneutrophilic inclusions were identified in the same region of northwestern Wisconsin and eastern Minnesota (*2*). Aside from the bacterial clusters, common features among these persons included fever, headache, myalgia, malaise, absence of skin rash, leukopenia, thrombocytopenia, and mild injury to the liver.

**Figure 1:**
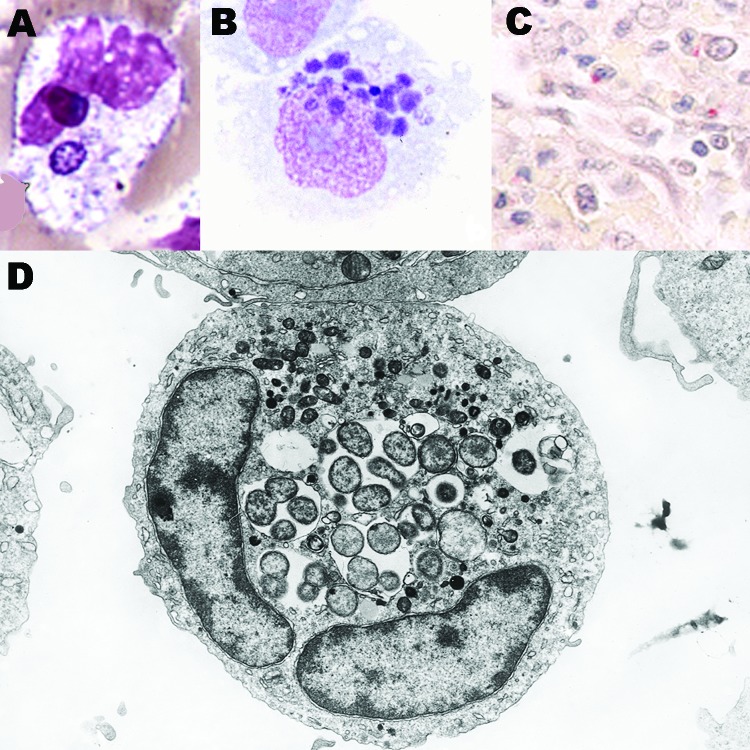
*Anaplasma phagocytophilum* in human peripheral blood band neutrophil (A. Wright stain, original magnification ×1,000), in THP-1 myelomonocytic cell culture (B, LeukoStat stain, original magnification, ×400), in neutrophils infiltrating human spleen (C, immunohistochemistry with hematoxylin counterstain; original magnification ×100), and ultrastructure by transmission electron microscopy in HL-60 cell culture (D; courtesy of V. Popov; original magnification ×21,960).

In 1994, through application of broad range molecular amplification and DNA sequencing, the causative agent was recognized as distinct from *E*. *chaffeensis*. The agent was initially named HGE agent (*1**,**2*), although morphologic and serologic studies indicated a close or identical relationship to the veterinary pathogens of neutrophils, *E*. *equi* and *E*. (*Cytoecetes*) *phagocytophila*. During the process of classification of the human agent, phylogenetic studies showed taxonomic disarray among organisms broadly referred to as ehrlichiae, and a careful reorganization now places those bacteria previously classified as *E*. *phagocytophila*, *E*. *equi*, and the HGE agent into a different genus as a single species, *A*. *phagocytophilum* (Figure 2) (*1**,**3*). The fallout from the reclassification of these organisms is the proposal for a complete revision of the families *Rickettsiaceae* and *Anaplasmataceae*. Under the proposed revision, the tribe structure of the *Rickettsiaceae* would be abolished, and species in the *Ehrlichieae* tribe would be assigned to the family *Anaplasmataceae*, with several placed into the genera *Ehrlichia* (*Cowdria ruminantium*), *Anaplasma* (*E*. *equi*, *E*. *phagocytophila*, HGE agent, *E*. *platys*, *E*. *bovis*), and *Neorickettsia* (*E*. *sennetsu* and *E*. *risticii*). The genera *Ehrlichia* and *Anaplasma* possess all pathogens in the family that are transmissible by ticks and that generally infect peripheral blood cellular elements, including leukocytes, platelets, and erythrocytes.

**Figure 2:**
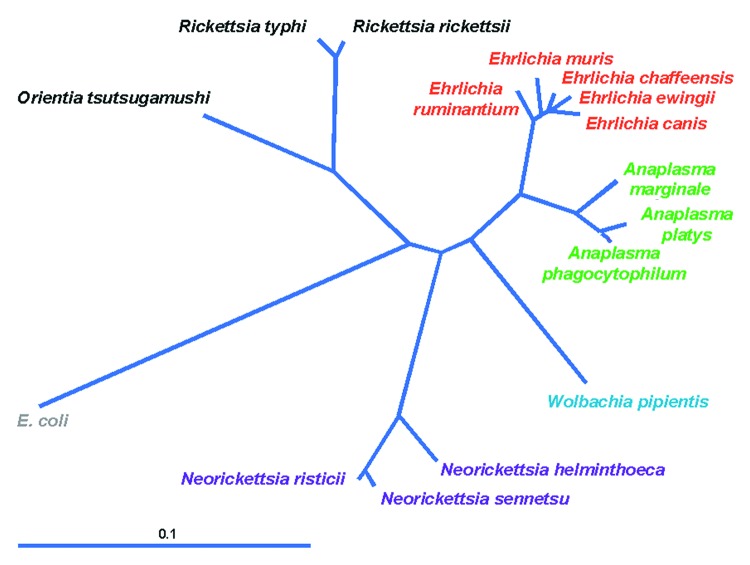
Current phylogeny and taxonomic classification of genera in the family *Anaplasmataceae*. The distance bar represents substitutions per 1,000 basepairs. *E*. *coli*, *Escerichia coli*.

HGA is increasingly recognized as an important and frequent cause of fever after tick bite in the Upper Midwest, New England, parts of the mid-Atlantic states, northern California, and many parts of Europe, all areas where *Ixodes* ticks bite humans (*4**–**6*). The ecology of *A*. *phagocytophilum* is increasingly understood. The bacterium is maintained in a transmission cycle with *Ixodes persulcatus* complex ticks, including *I. scapularis* in the eastern United States, *I*. *pacificus* in the western United States, *I*. *ricinus* in Europe, and probably *I*. *persulcatus* in parts of Asia. Tick infection is established after an infectious blood meal, and the bacterium is transstadially but not transovarially passed (*3*). The major mammalian reservoir for *A*. *phagocytophilum* in the eastern United States is the white-footed mouse, *Peromyscus leucopus*, although other small mammals and white-tailed deer (*Odocoileus virginianus*) can also be infected. White-footed mice have transient (1–4 weeks) bacteremia; deer are persistently and subclinically infected. Human infection occurs when humans impinge on tick–small mammal habitats (*4**–**7*).

HGA is clinically variable, but most patients have a moderately severe febrile illness with headache, myalgia, and malaise. Among 10 clinical studies that describe the findings in HGA across North America and Europe and that comprise up to 685 patients (Table), the most frequent manifestations are malaise (94%), fever (92%), myalgia (77%), and headache (75%); a minority have arthralgia or involvement of the gastrointestinal tract (nausea, vomiting, diarrhea), respiratory tract (cough, pulmonary infiltrates, acute respiratory distress syndrome [ARDS]), liver, or central nervous system (*4**–**7*). Rash is observed in 6%, although no specific rash has been associated with HGA and co-infection with *Borrelia burgdorferi*, which can cause simultaneous erythema migrans, is not infrequent. Frequent laboratory abnormalities identified in up to 329 patients include thrombocytopenia (71%), leukopenia (49%), anemia (37%), and elevated hepatic transaminase levels (71%).

**Table:** Metaanalysis of clinical manifestations and laboratory abnormalities in patients with human granulocytic anaplasmosis*

Characteristics	All			North America		Europe	
	Median %†	Mean %	n‡	Mean %	n	Mean %	n
Symptom or sign							
Fever	100	92	480	92	448	98	66
Myalgia	74	77	514	79	448	65	66
Headache	89	75	378	73	289	89	66
Malaise	93	94	90	96	271	47	15
Nausea	44	38	256	36	207	47	49
Vomiting	20	26	90	34	41	19	49
Diarrhea	13	16	90	22	41	10	49
Cough	13	19	260	22	207	10	49
Arthralgias	58	46	497	47	448	37	49
Rash	3	6	685	6	289	4	53
Stiff neck	11	18	22	22	18	0	4
Confusion	9	17	211	17	207	0	4
Laboratory abnormality							
Leukopenia	38	49	329	50	282	47	47
Thrombocytopenia	71	71	329	72	282	64	47
Elevated serum AST or ALT§	74	71	170	79	123	51	47
Elevated serum creatinine	15	43	72	49	59	0	13

*Data from references 5, 6, 8–15.

†Median percentage of patients with feature among all reports.

‡Number of patients with data available for metaanalysis.

§AST, aspartate aminotransferase; ALT, alanine aminotransferase.

Recent seroepidemiologic data suggest that many infections go unrecognized, and in endemic areas as much as 15% to 36% of the population has been infected (*16**,**17*). In Wisconsin, the yearly incidence of HGA from 1990 to 1995 was as high as 58 cases/100,000 in 1 county (Lyme disease incidence in the same region was 110 cases/100,000) (*5*). The overall yearly Connecticut incidence rate from 1997 to 1999 was 24 to 51 cases/100,000 population (*18*). Symptomatic infection in Europe appears to be rare; 66 cases have been reported, despite a median seroprevalence rate of 6.2% among 35 published reports, with rates as high as 21% in some European studies. Similarly, the median infection prevalence in European *I*. *ricinus* ticks is 3% (45 publications), a figure close to that observed among North American *I*. *scapularis* and *I*. *pacificus* ticks (median 4.7% among 42 publications).

What is unclear from these data is whether the discrepancy between the seroprevalence and symptomatic rate results from underdiagnosis of infection, asymptomatic serologic reactions, or even infections that produce cross-reactive serologic responses. In any case, symptomatic infection can occur often in tick-endemic regions and varies in severity from mild, self-limited fever to death. Severity sufficient for hospitalization is observed in half of symptomatic patients and is associated with older age, higher neutrophil counts, lower lymphocyte counts, anemia, the presence of morulae in leukocytes, or underlying immune suppression (*5*). Approximately 5%–7% of patients require intensive care, and at least 7 deaths have been identified (*2**,**4**,**5**,**7**,**19*), in which delayed diagnosis and treatment were risk factors. Severe complications include a septic or toxic shock–like syndrome, coagulopathy, atypical pneumonitis/acute respiratory distress syndrome (ARDS), acute abdominal syndrome, rhabdomyolysis, myocarditis, acute renal failure, hemorrhage, brachial plexopathy, demyelinating polyneuropathy, cranial nerve palsies, and opportunistic infections. At least 3 of the deaths resulted from opportunistic fungal or viral infections or hemorrhage that occurred immediately after HGA. In 2 cases, the patients had reasons for preexisting immunocompromise, which suggests that an intact immune system is important for recovery and that HGA further antagonizes immune dysfunction (*2**,**4**,**5**,**7*). Unlike results of animal observations (*20*), no evidence has shown *A*. *phagocytophilum* persistence in humans.

## Pathology of *A*. *phagocytophilum* Infections in Humans

Few histopathologic studies of HGA have been conducted. Of 7 patients with fatal cases, 3 died from opportunistic infections (*2**,**4**,**5**,**7*), including exsanguination after ulcerative *Candida* esophagitis, ulcerative herpes simplex virus esophagitis with cryptococcal pneumonia, and invasive pulmonary aspergillosis. In 2 other deaths, the patients experienced myocarditis (likely viral) or generalized lymphadenopathy and mononuclear phagocyte system activation.

The pathologic changes in humans include perivascular lymphohistiocytic inflammatory infiltrates in multiple organs, hepatitis with infrequent apoptoses, normocellular bone marrow, mild lymphoid depletion, mononuclear phagocyte hyperplasia in spleen and lymph nodes, and, rarely, splenic necrosis. Hemophagocytosis is observed in bone marrow, liver, and spleen. Vasculitis has not been observed (*4*). By immunohistochemical tests, *A*. *phagocytophilum* is rarely identified; organisms were abundant in only 1 patient who died, rare in 2 patients, and not identified in 2 patients (*2**,**4**,**5**,**19*). Infected neutrophils are not generally associated with pathologic lesions, which suggests alternative mechanisms that do not involve direct bacteria-mediated injury.

Opportunistic infections and inflammatory changes in humans are not unexpected because similar findings occur in animals (*19**,**21*). In fact, tickborne fever (ruminant granulocytic anaplasmosis) induces diminished CD4 and CD8 peripheral blood counts, impaired mitogenic responses, impaired antibody responses, impaired neutrophil emigration, and defective phagocytosis and intracellular killing. Such in vitro findings are supported by clinical observations, which document that bacterial, fungal, and viral infections are frequent and generally worse in animals with tickborne fever (*20*). Disseminated staphylococcal infections that occur with tickborne fever kill ≈2% of field-raised sheep in the United Kingdom (*20*); louping ill, a tickborne viral encephalitis of goats is self-limited unless it occurs in conjunction with tickborne fever, when it is often fatal (*20*); bacterial and fungal secondary infections are more frequent in *A*. *phagocytophilum*–infected horses (*21*). A likely interpretation is that *A*. *phagocytophilum* is associated with perturbations in host inflammatory and immune system function. Impaired early inflammatory responses that might be induced by *A*. *phagocytophilum* could contribute to the pathogenesis of HGA, and early initiation of proinflammatory and immune responses depend on functionally competent neutrophils and mononuclear phagocytes.

## Pathogenesis of *A*. *phagocytophilum* Infections

*Anaplasma* species are small (0.2–1.0 μm in diameter) obligate intracellular bacteria with a gram-negative cell wall (*4*), but lack lipopolysaccharide biosynthetic machinery (*22*). The bacteria reside in an early endosome, where they obtain nutrients for binary fission and grow into a cluster called a morula (Figure 1). Recent genomic studies demonstrated a type IV secretion apparatus, which could facilitate transfer of molecules between the bacterium and the host (*23**,**24*). *A*. *phagocytophilum* prefers to grow in myeloid or granulocytic cells and has been propagated in human HL-60 and KG-1 promyelocytic leukemia cells, THP-1 myelomonocytic cells, endothelial cell cultures, and tick cell cultures (*3*). HL-60 cells induced to differentiate into neutrophil-like cells cease to divide but enhance *A*. *phagocytophilum* growth. When differentiated into monocytic cells, HL-60 cells no longer support *A*. *phagocytophilum* growth.

*A*. *phagocytophilum* binds to fucosylated and sialylated scaffold proteins on neutrophil and granulocyte surfaces (*25*). The most studied ligand is PSGL-1 (CD162) to which the bacterium adheres at least in part through 44-kDa major surface protein-2 (Msp2) (*26*). Msp2 is probably part of an "adhesin complex" involving Msp2 oligomers with other membrane proteins. After internalization of bacteria, the endosome ceases to mature and does not accumulate markers of late endosomes or phagolysosomes (*27*). As a result, the vacuole does not become acidified or fuse to lysosomes. *A*. *phagocytophilum* divides until cell lysis or bacteria are discharged to infect other cells.

The range of described *A*. *phagocytophilum* proteins is limited, although the genome sequence should assist in defining bacterial structure and function. The most abundant protein in *A*. *phagocytophilum* is Msp2, encoded by a multigene family of at least 22 paralogs in the Webster strain genome and 52 or more paralogs in the HZ strain genome (*28*). Antigenic diversity among *A*. *phagocytophilum* strains from different regions is increased by *msp2* gene conversion. Diversity is assumed to be driven by immune selection and may play an important role in persistence among reservoir hosts, but restricted *msp2* transcription and Msp2 expression over many passages and in tick cells suggest selection by fitness for new niches, a finding underscored by Msp2's role as an adhesin (*26**,**28*).

Aside from *msp2*, *ankA* is the most actively studied *A*. *phagocytophilum* component (*24**,**29*). This gene encodes a 153–160 kDa protein with at least 11 N-terminal ankyrin repeats and a C-terminus with several tandem repeats but no homology with other proteins. AnkA sequences are diverse according to geographic origin, with relative conservation among North American strains and diversity among European bacteria. Whether *ankA* diversity relates to severity is not known. An interesting observation regarding AnkA is its localization, where it forms a complex with chromatin in the infected granulocyte cell nucleus. Although little is known about whether AnkA affects *A*. *phagocytophilum* survival or pathogenesis, it is currently the only protein of *A*. *phagocytophilum* known to be secreted by the bacterium, that passes through the bacterial and vacuolar membrane (presumably by the *A*. *phagocytophilum* type IV secretion mechanism [23]), through the cytoplasm and nuclear membrane, to find a nuclear target. Within the nucleus of infected neutrophils or HL-60 cells, AnkA binds nuclear proteins and complexes to AT-rich nuclear DNA that lacks specific conserved sequences (*29*). Its mere presence in the nucleus of a cell in which gene transcription appears to be altered by infection compels further investigation of a direct pathogenetic role in regulation of eukaryotic gene expression.

### Animal Models and Immunopathogenicity

The discrepancy between bacterial load and histopathologic changes with HGA suggests that disease relates to immune effectors that inadvertently damage tissues. In vivo human cytokine responses are dominated by interferon-γ (IFNγ) and interleukin-10 (IL-10), but lack tumor necrosis factor α (TNFα), IL-1β, and IL-4 (*30*), which suggests a role for macrophage activation in recovery and disease. A murine model shows a cytokine profile similar to that in humans and reproduces histopathologic lesions in infected humans, horses, and dogs (*19*). In this model, bacterial load peaks at day 7 and rapidly declines; IFNγ peaks at day 10 and also declines in parallel. However, histopathologic injury, minimal at days 7–10, peaks by day 14, and then resolves. This pattern suggests a role for IFNγ in histopathology and restriction of infection, which is confirmed since histopathologic lesions do not develop in IFNγ knockout mice, but the mice have a 5- to 8-fold increase in bacteremia levels (*31*). In contrast, IL-10 knockout mice, which poorly restrict INFγ production, do not have increased bacteremia levels, yet histopathologic lesions are significantly worse than controls. The mechanisms of bacterial growth restriction seem clearly related to INFγ production, but the role of NOS2 (iNOS) in this process is unresolved. Activation of innate immune responses through TLR2, TLR4, MyD88, TNFα, and CYBB does not contribute to control of *A*. *phagocytophilum*. Several murine models show no correlation between histopathologic injury and bacterial load. Likewise, infection of TLR2-, TLR4-, MyD88-, TNFα-, and CYBB-knockout mice does not affect bacterial burden, yet abrogates inflammatory tissue lesions. Such findings support an immune triggering role for *A*. *phagocytophilum* as a mechanism for disease.

While IFNγ and IL-10 are key markers or effectors of injury with *A*. *phagocytophilum* infection, their source is unclear. Infection of neutrophils and HL-60 cells differentiated into neutrophil-like cells produces striking quantities of CXC and CC chemokines, including IL-8, RANTES, MIP1α, MIP1β, and MCP-1, but not IFNγ, IL-10, TNFα, IL-1β, or IL-4 (*32*), suggesting that *A*. *phagocytophilum* infection partially activates neutrophils. Akkoyunlu et al. demonstrated a decreased bacteremia with antibody blockade of chemokine receptors (CXCR2) and in CXCR2 knockout mice (*33*). This presumably provides a survival advantage to the bacterium by recruitment of new neutrophil host cells, increasing the blood concentrations of infected cells that can be acquired by tick bite. In spite of the increased bacteremia, no increase in histopathologic lesions is noted, confirming previous studies. The disadvantage of chemokine production to the host is that recruitment of inflammatory cells that are activated could produce IFNγ-induced inflammation, leading to damage to tissues.

### Neutrophil Functional Changes with *A*. *phagocytophilum* Infection

Other notable alterations of neutrophil function and physiology are observed with *A*. *phagocytophilum* infection. *A*. *phagocytophilum* survives its initial encounter by detoxifying superoxide produced by neutrophil phagocyte oxidase assembly, perhaps by virtue of bacterial superoxide dismutase (*23**,**34*). Although not yet shown in infected neutrophils, infected HL-60 cells are unable to generate respiratory bursts because of reduced transcription of components of phagocyte oxidase, including gp91^phox^ and Rac2 (*35**,**36*). Although this defect seems limited to the infected neutrophils and is a major mechanism that permits intracellular infection, the reduction in phagocyte oxidase may have other effects, including a reduction in local regulation of inflammation. This results from the inability of phagocyte oxidase to degrade inflammatory mediators such as leukotrienes, complement, and perhaps other components. Another normal function of neutrophils is apoptosis, which regulates inflammation by programmed cell death of activated neutrophils usually within 24 to 48 hours. The induction of apoptosis by *A*. *phagocytophilum*–infected neutrophils is delayed ≈24 hours (*37*) and also relates to maintained transcription of *bcl2* family genes and stabilization of the mitochondrial pathway that ultimately prevents procaspase 3 processing (*37*).

Infection by *A*. *phagocytophilum* results in significant disruption of normal neutrophil function, including endothelial cell adhesion and transmigration, motility, degranulation, respiratory burst, and phagocytosis. *A*. *phagocytophilum*–infected neutrophils and HL-60 cells are inhibited from binding to systemic and brain microvascular endothelial cells, even under conditions of low shear force (*38*). The adhesion defect results from the shedding of neutrophil PSGL-1 and L-selectin, which mediate the critical first step in inflammatory cell recruitment. This inhibited recruitment occurs despite the rapid mobilization of surface β2-integrins (CD11b/CD18) and ICAM-1 (CD54), which ordinarily mediate the second phase of tight endothelial-cell binding. Thus, *A*. *phagocytophilum*–infected neutrophils are inhibited from transmigrating endothelial cell barriers in spite of stimulated motility. Selectin "shedding" occurs because infected cells degranulate, including an EDTA-inhibitable sheddase (metalloprotease), β2-integrins, CD66b, and other inflammatory components such as matrix metalloproteases, which includes gelatinase (MMP9) (*38**,**39*). Engagement of opsonophagocytosis receptors and degranulation are usually accompanied by rapid cell death (apoptosis), but with *A*. *phagocytophilum*, degranulation occurs over a prolonged period, potentially exacerbating inflammation, especially with delayed apoptosis of infected neutrophils (*36**,**39**,**40*). After recruitment, chemotactic migration, and activation for respiratory burst, neutrophils are then activated for phagocytosis; however, this function is inhibited in vivo and in vitro, perhaps in part resulting from alterations of *rac2* expression and loss of important surface receptors (*40*). Altogether, the activated-deactivated phenotype of the *A*. *phagocytophilum*–infected neutrophil may benefit the bacterium by increasing concentrations of infected cells in the peripheral blood that are unresponsive to tissue recruitment and may have a prolonged lifespan. However, the cost to the host includes activation of neutrophils to participate in proinflammatory reactions while they are unable to act as microbicidal effectors or regulators of inflammation.

## Conclusions

Investigators of novel intracellular bacteria often address unanswered questions by investigating processes shared with other bacteria or bacterial processes, or by investigating differences that have allowed the unique niche to become occupied. Since *A*. *phagocytophilum*, along with *E*. *ruminantium*, *E*. *ewingii*, and *Chlamydophila pneumoniae* are the only known bacteria to survive and propagate within neutrophils, it seems most relevant that investigation should focus on adaptations permissive for neutrophil infection. What is clear is that this new tickborne infection has a great capacity to infect and cause disease in humans while maintaining a persistent subclinical state in animal reservoirs. The disease processes appear to be immune and inflammatory in nature, not directly related to pathogen burden, and result by the triggering of a detrimental and poorly regulated host response. Recent investigations have provided important phenotypic data on the range of functional changes among *A*. *phagocytophilum*–infected neutrophils and identified several compelling targets for study of fundamental pathogenetic processes. Important areas that still need intense study include the bacterial triggers of host innate and inflammatory response and the molecular and cellular mechanisms by which *A*. *phagocytophilum* influences cell function and ultimately causes injury to host cells, tissues, and organs. Perhaps by developing a more comprehensive understanding of the basic mechanisms underlying *A*. *phagocytophilum*–neutrophil/host interactions, we can appropriately target strategies for control and management.
